# Visceral fat mass: is it the link between uric acid and diabetes risk?

**DOI:** 10.1186/s12944-017-0532-4

**Published:** 2017-07-24

**Authors:** Neda Seyed-Sadjadi, Jade Berg, Ayse A. Bilgin, Ross Grant

**Affiliations:** 1 School of Medical Sciences, Faculty of Medicine, University of New South Wales, Sydney, NSW Australia; 2 Australasian Research Institute, Sydney Adventist Hospital, 185 Fox Valley Road, Wahroonga, Sydney, NSW 2076 Australia; 3 Department of Statistics, Macquarie University, Sydney, NSW Australia; 4 Sydney Adventist Hospital Clinical School, University of Sydney, Sydney, NSW Australia

**Keywords:** Diabetes, Uric acid, Glucose, HbA1c, Triglyceride, Inflammation, BMI, Android fat, Gynoid fat, Visceral fat mass

## Abstract

**Background:**

Uric acid (UA) has been suggested as a novel risk factor for diabetes. However, its definite role in this prevalent disease is still the subject of much discussion because it is always accompanied with other major risk factors such as obesity and high visceral adiposity. In order to clarify the role of UA in diabetes, this study aimed to investigate the associations between plasma UA and fasting plasma glucose, HbA1c, lipid profile and inflammatory markers after accounting for the contribution of other diabetes risk factors such as BMI and VAT fat mass.

**Methods:**

In the present cross-sectional study, 100 non-diabetic middle-aged males (*n* = 48) and females (*n* = 52) were recruited. Central fat distribution measures including android to gynoid fat ratio, VAT and subcutaneous adipose tissue (SAT) fat mass were determined using dual-energy X-ray absorptiometry (DXA). Biochemical analysis was done using methods well established for clinical and research laboratories. Multiple linear regression analysis was performed to analyse the association between plasma UA and the biochemical and central fat distribution measures.

**Results:**

UA was positivly associated with body mass index (BMI) (*r* (98) = 0.42, *P* ≤ 0.001), android to gynoid fat ratio (*r* (98) = 0.62, *P* ≤ 0.001) and VAT fat mass (*r* (96) = 0.55, *P* ≤ 0.001). UA was also positively associated with plasma glucose (*r* (98) = 0.33, *P* ≤ 0.001), hemoglobin A1c (*r* (93) = 0.25, *P* = 0.014), plasma triglyceride (*r*_*s*_ (95) = 0.40, *P* ≤ 0.001), HDL cholesterol (*r* (98) = − 0.61, *P* ≤ 0.001) and CRP (*r*_*s*_ (98) = 0.23, *P* = 0.026). However, these associations were no longer significant after accounting for BMI or/and VAT fat mass. No significant association was observed between UA and SAT fat mass (*r* (97) = 0.02, *P* ≥ 0.05), Total cholesterol (*r* (98) = 0.03, *P* ≥ 0.05), LDL cholesterol (*r* (98) = 0.13, *P* ≥ 0.05), TNF-α (*r* (97) = 0.12, *P* ≥ 0.05) and IL-6 (*r* (96) = −0.02, *P* ≥ 0.05).

**Conclusion:**

Results from this study suggest, for the first time, that the association between plasma UA and glucose in a non-diabetic population is not direct but rather dependent on VAT fat mass.

## Background

The predicted increase in diabetes prevalence from 366 million in 2011 to an estimated 552 million by 2030 highlights the critical need for preventive strategies to this ubiquitous degenerative disease [1]. Identifying the risk factors for developing diabetes is crucial for prevention. In this context, uric acid (UA), the end product of purine metabolism, has been proposed as a novel risk factor for diabetes [2–4]. A meta-analysis of cohort studies reported an overall 17% increase in diabetes risk with every 0.059 mmol/L serum UA elevation [4].

However, recognition of high serum UA as a risk factor for diabetes has been a matter of debate for some time with consensus on the final mechanism still unresolved. Even though some studies have shown a positive correlation between serum UA and levels of fasting plasma glucose (FPG) [5, 6], glycated hemoglobin A1c (HbA1c) [6], triglyceride (TG) [5, 7, 8], and inflammatory markers [9] and inverse correlations with high density lipoprotein cholesterol (HDL-C) levels [7, 8], other studies have actually observed an inverse association between serum UA and diabetes [10, 11]. More importantly, whether UA exerts its effect independent of other established diabetes risk factors is still not known. Although many previous studies have accounted for a substantial range of potential metabolic confounders in their design [12, 13], there is still one major risk factor with strong correlation with UA that has not been accounted for.

UA levels have been shown to be strongly correlated with body mass index (BMI) (14), android to gynoid fat ratio (15) and more specifically, visceral adipose tissue (VAT), a component of android fat mass (14, 15). There is plenty of evidence suggesting that obesity (BMI ≥ 25) and high android to gynoid fat ratio are important features of insulin resistance and diabetes [14–16]. In particular, excess accumulation of (VAT) fat mass has been reported to be associated with insulin resistance by release of free fatty acids, hormones, and inflammatory proteins and chemokines [17]. There is also evidence that subcutaneous adipose tissue (SAT), another component of android fat, impacts the development of obesity related insulin resistance by releasing fatty acids [18].

The intercorrelations among obesity, VAT fat mass, serum UA and diabetes make it difficult to determine whether or not an elevated serum UA level is an independent causative risk factor or rather a dependent marker subordinate to the pathological changes occurring in diabetes. A better understanding of the relationships between plasma UA levels and these risk factors of diabetes is necessary to clarify the definite role of UA in diabetes development. Even though the role of BMI and waist circumference has been accounted for in many studies [12, 13]; the specific role of VAT fat mass has not yet been considered. Furthermore, the relationship between diabetes risk factors and serum UA levels in a non-diabetic population is still less known and needs further investigation. In this study, we investigated whether plasma UA concentration was associated with diabetes risk factors such as FPG, HbA1c, lipid profile and inflammatory markers in middle-aged non-diabetics independent of BMI and VAT fat mass. To the best of our knowledge, this is the first examination of non-diabetic males and females for these associations taking in to account VAT fat mass.

## Methods

### Participants

In this cross-sectional study male (*n* = 48) and female (*n* = 52) participants aged between 40 and 75 years were recruited at Sydney Adventist hospital and the university of New South Wales campuses. Participants were non-diabetics and considered in general good health. Blood collection and body scanning for central fat distribution analysis were performed on the same day in a fasted state (about 12 h) after obtaining written informed consent. Ethical approval of the study was obtained from the Adventist HealthCare Limited Human Research Ethics Committee, Sydney Adventist Hospital, Australia (HREC number: 2013–022).

### Biochemical analysis

The measurements of plasma UA, FPG, HbA1c, total cholesterol (TC), Low density lipoprotein cholesterol (LDL-C), HDL-C and TG and C-reactive protein (CRP) were performed by the Sydney Adventist Hospital pathology laboratory using methods well established for clinical laboratories. Briefly, fasting plasma UA, FPG, TG, TC, and HDL-C levels were determined by the enzymatic method on a Roche/Hitachi cobas c system. LDL-C levels were calculated by the Friedewald eq. [19]. Plasma HbA1c concentration was measured by ion-exchange high-performance liquid chromatography (HPLC) on the D-100 hemoglobin testing system (Bio-Rad Laboratories, Hercules, CA, USA). Plasma CRP was measured by Immunoturbidimetric assay on a Roche/Hitachi cobas c system. Plasma tumor necrosis factor- α (TNF-α) and interleukin-6 (IL-6) levels were quantitated using the MILLIPLEX® MAP Human High Sensitivity T Cell Magnetic Bead Panel immunoassay (Merck KGaA, Darmstadt, Germany). Estimated Glomerular filtration rate (eGFR) was calculated using the simplified Modification of Diet in Renal Disease study equation: GFR (ml/min/1.73m^2^) = 186 × (serum creatinine level [mg/dl])-1.154 × (age)-0.203× [0.742, if female] × [1.212, if black] [20, 21].

### Central fat distribution analysis

Dual-energy X-ray absorptiometry (DXA) method was used to measure central fat distribution by a Lunar iDXA (GE Healthcare, Madison, WI, USA) with automatic total body scan mode and enCORE software (version 16, GE Healthcare, Madison, WI, USA). All participants were scanned by trained operators according to the standard methods. Quality control scans were obtained daily using the manufacturer supplied calibration phantom. All metal items were removed from the participant before scanning. The participants were asked to change into a standard cloth gown and were correctly centred on the scanning table in a supine position with arms at sides slightly separated from the trunk and the palms facing the thighs.

The regions of interest (ROIs) for regional body composition were automatically drawn by the software according to anatomical landmarks and then double-checked with a trained operator for obtaining the best position. Two ROIs were considered in this study: android and gynoid ROIs. Android region (roughly 10 cm in height) was defined as a portion of the abdomen included between pelvis cut line (the line joining the two superior iliac crests) and the 20% of the distance between pelvis cut line and neck cut line. Gynoid region (two times the android region) was defined as a portion of legs beginning at a distance of 1.5 times android ROI height below pelvis cut line and directed caudally up to a distance double that of the height of the Android ROI [22].

Central fat distribution pattern was assessed by the android to gynoid fat ratio that is the ratio of android fat (kg) divided by gynoid fat (kg). VAT fat mass was computed automatically for the android ROI by CoreScan algorithm, a new software option in enCORE for the estimation of visceral fat mass within the android region [23]. In the android ROI, SAT was calculated manually by subtracting VAT from total android fat mass.

Anthropometric measures were obtained using a standardized protocol. Weight (kg) and height (cm) were measured to the nearest 0.5 kg and 0.1 cm, with participants wearing a cloth gown and without shoes. BMI was calculated as weight in kilograms divided by the square of the height in meters.

### Statistical analysis

Statistical analysis was performed using SPSS version 23 for Windows. Values are presented as means ± standard deviations. The Kolmogorov-Smirnov and Shapiro-Wilk tests were used to test the normality of the variables. After checking graphical displays and applying appropriate statistical rules, outliers were removed for UA (*2), TG (*3), VAT (*3) and SAT fat mass (*1), CRP (*3), IL-6 (*1) and TNF-α (*1). Correlations between variables were determined using Pearson’s (*r* (n)) or Spearman’s (*r*_*s*_ (n)) correlation coefficients, as appropriate. The Student’s t-test or Mann-Whitney U-test were used, as appropriate, to determine differences in the means of continuous variables.

Finally, multiple linear regression analysis was performed to determine the association between the plasma UA and FPG, HbA1c, TG, HDL-C, CRP, BMI, and abdominal adiposity measures including android to gynoid fat ratio and the VAT fat mass, after adjustment for age, gender, eGFR, BMI and/or VAT fat mass. The Levene’s Test of Equality was applied to check homogeneity of variances between groups. If the variances of the groups were either not homogenous and/or normality tests for the variables/multiple linear regression models were significant then further examination with graphical displays was performed and where needed base-10 log-transformed means, square roots or reciprocals were used or the association was analysed separately for each group. *P* values less than 0.05 were considered statistically significant.

## Results

Among a total of 100 subjects, 48 were males and 52 were females. Clinical characteristics of the study participants are shown in Table 1. The mean age was 56.0 ± 8.8 years. The mean FPG and HbA1c levels were 5.1 ± 0.6 mmol/L and 34.6 ± 3.5 mmol/mol, respectively. There were no significant differences between genders for either FPG or HbA1c levels. The mean plasma UA levels were 0.3 ± 0.07 mmol/L. Average UA levels were significantly higher for males than females. There were no significant differences between genders for plasma levels of TG, LDL-C, TC, eGFR, and inflammatory markers (i.e. CRP, TNF- α, and IL-6). However, as expected, HDL-C levels were significantly higher in females than males. The mean values for BMI, android to gynoid fat ratio, VAT and SAT fat mass were 26.2 ± 4.6 kg/m^2^, 1.1 ± 0.3, 953.1 ± 798.3 g and 1314.4 ± 673.8 g, respectively. The mean BMI was not statistically significant between genders. However, there were statistically significant differences between genders for android to gynoid fat ratio, VAT and SAT fat mass values. Android to gynoid fat ratio and VAT fat mass were significantly higher in males than females, while SAT fat mass was significantly higher in females than in males.

**Table 1:** Participant characteristics according to gender

	Total (Mean ± SD) (*n* = 100)	Male (Mean ± SD) (*n* = 48)	Female (Mean ± SD) (*n* = 52)	*P* value
Age (years)	55.98 ± 8.83	56.60 ± 9.40	55.40 ± 8.33	NS^a^
FPG (mmol/L)	5.11 ± 0.60	5.19 ± 0.70	5.03 ± 0.49	NS^b^
HbA1c (IFCC) (mmol/mol)	34.60 ± 3.55	34.83 ± 3.86	34.38 ± 3.25	NS^b^
UA (mmol/L)	0.32 ± 0.07	0.35 ± 0.07	0.28 ± 0.06	*P* < 0.001^b^
TG (mmol/L)	1.23 ± 0.62	1.38 ± 0.75	1.09 ± 0.45	NS^a^
LDL-C (mmol/L)	3.24 ± 0.99	3.32 ± 0.9	3.17 ± 1.05	NS^a^
HDL–C (mmol/L)	1.65 ± 0.51	1.36 ± 0.31	1.92 ± 0.50	*P* < 0.001^a^
TC (mmol/L)	5.51 ± 1.15	5.41 ± 1.08	5.60 ± 1.21	NS^a^
CRP (mg/L)	1.42 ± 1.30	1.33 ± 1.28	1.51 ± 1.32	NS^a^
IL-6 (pmol/L)	2.67 ± 1.96	2.57 ± 1.99	2.75 ± 1.94	NS^a^
TNF-α (pmol/L)	8.13 ± 2.41	8.31 ± 2.48	7.97 ± 2.35	NS^a^
Estimated GFR (mL/min/1.73 m2)	80.36 ± 9.91	81.34 ± 9.12	79.48 ± 10.58	NS^a^
BMI (kg/m^2^)	26.24 ± 4.58	26.31 ± 4.02	26.17 ± 5.11	NS^a^
Android to gynoid fat ratio	1.13 ± 0.32	1.32 ± 0.30	0.95 ± 0.23	*P* < 0.001^b^
VAT fat mass (g)	953.12 ± 798.26	1277.25 ± 870.96	672.63 ± 610	*P* < 0.001^a^
SAT fat mass (g)	1314.44 ± 673.77	1009.14 ± 476.72	1601.78 ± 708.85	*P* < 0.001^a^

^a^Comparisons made using Mann-Whitney U Test

^b^Comparisons made using the Independent T Test

### Associations between BMI, and central fat distribution parameters (android to gynoid fat ratio, VAT and SAT fat mass) and plasma TG and FPG

As expected, a statistically positive association was observed between FPG and BMI (*r* (100) = 0.31, *P* ≤ 0.01). This association remained statistically significant after controlling for age, gender and eGFR (t (94) = 2.92, *P* = 0.004, *R* ^2^ = 0.14). FPG was also significantly associated with android to gynoid fat ratio (*r*(100) = 0.384, *P* ≤ 0.001). This association remained statistically significant after controlling for age, gender and eGFR (t (94) = 3.73, *P* ≤ 0.001, *R* ^2^ = 0.18).

A statistically significant association was also observed between FPG and VAT fat mass (*r* (97) = 0.32, *P* ≤ 0.001) that remained statistically significant after controlling for age, gender and eGFR (t (91) = 2.84, *P* ≤ 0.01, *R* ^2^ = 0.13). No association was observed between FPG and SAT fat mass (*r* (99) = 0.107, *P* ≥ 0.05). Also, there was a statistically significant association between FPG and TG (*r* (97) = 0.46, *P* ≤ 0.001). Since the residuals for the linear regression model were not normally distributed when gender was in the equation, the association between plasma TG and FPG was analysed separately for each gender. The results showed that after controlling for age, eGFR and BMI there was a statistically significant association between FPG and plasma TG in both males (t (39) = 2.18, *P* = 0.035, *R* ^2^ = 0.26) and females (t (47) = 3.58, *P* ≤ 0.001, *R* ^2^ = 0.24).

### Associations between plasma UA, FPG and plasma HbA1c

**Fig. 1:**
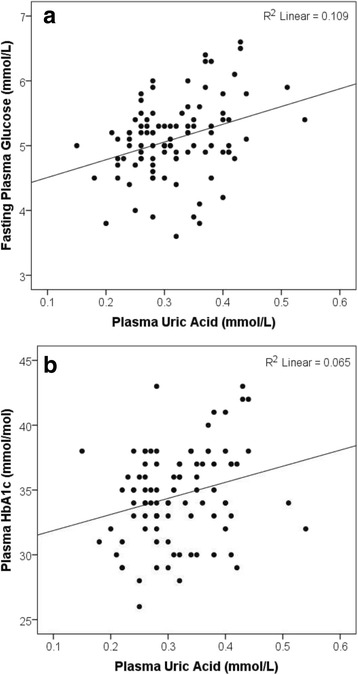
Associations between plasma uric acid and **a** Fasting plasma glucose **b** Plasma HbA1c

There was a statistically significant positive association between plasma UA and FPG (*r* (98) = 0.33, *P* ≤ 0.001) (Fig. 1). This association remained statistically significant after adjustment for age, gender and eGFR (*t* (92) = 2.67, *P* ≤ 0.01, *R* ^2^ = 0.14). However, it did not remain significant after further adjustment for BMI (*t* (91) = 1.76, *P* ≥ 0.05, *R* ^2^ = 0.15) or VAT fat mass (t (89) = 1.64, *P* ≥ 0.05, *R* ^2^ = 0.15).

A statistically significant positive association was observed between plasma UA and plasma HbA1c (*r* (93) = 0.25, *P* = 0.014) (Fig. 1). This association remained statistically significant after adjustment for age, gender and eGFR (*t* (87) = 2.12, *P* = 0.036, *R* ^2^ = 0.08). However, as with FPG, the association did not remain significant after further adjustment for BMI (*t* (86) = 1.37, *P* ≥ 0.05, *R* ^2^ = 0.08) or VAT fat mass (t (84) = 1.09, *P* ≥ 0.05, *R* ^2^ = 0.08).

### Associations between plasma UA, BMI and central fat distribution parameters (android to gynoid fat ratio, VAT and SAT fat mass)

**Fig. 2:**
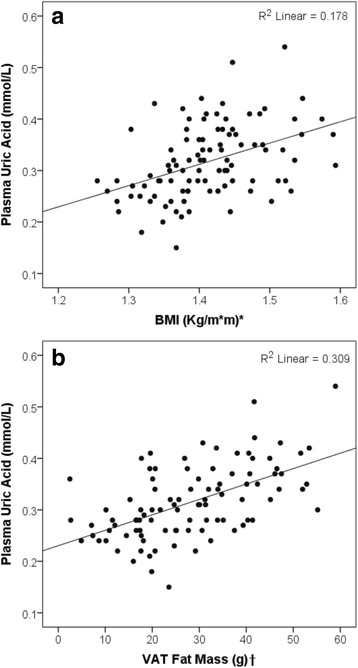
Associations between plasma uric acid and **a** BMI **b** VAT fat mass. *Data represented as base-10 log-transformed BMI, †data represented as Square root VAT fat mass

A statistically significant positive association was observed between plasma UA and BMI (*r* (98) = 0.42, *P* ≤ 0.001) (Fig. 2). This association remained statistically significant after controlling for age, gender and eGFR (*t* (92) = 4.96, *P* ≤ 0.001, *R* ^2^ = 0.44). However, it did not remain significant after adding and adjusting for VAT fat mass (t (89) = 1.26, *P* ≥ 0.05, *R* ^2^ = 0.44). This model was able to account for 44% of variability in mean plasma UA levels.

There was a statistically significant positive association between plasma UA and android to gynoid fat ratio (*r* (98) = 0.62, *P* ≤ 0.001). This association remained statistically significant after controlling for age, gender, eGFR and BMI (*t* (91) = 2.44, *P* = 0.017, *R* ^2^ = 0.47), but no longer remained significant after adjusting for VAT fat mass (t (89) = 1.48, *P* ≥ 0.05, R^2^ = 0.45).

A positive association between plasma UA and VAT fat mass was also observed (*r* (96) = 0.55, *P* ≤ 0.001) (Fig. 2). This association remained statistically significant after controlling for age, gender and eGFR (*t* (90) = 4.96, *P* ≤ 0.001, *R* ^2^ = 0.44), and even after including BMI in the model (t (89) = 2.33, *P* = 0.022, *R* ^2^ = 0.44). This model was able to account for 44% of variability in mean UA levels.

Plasma UA levels were significantly different between groups with BMI < 25 and BMI ≥ 25 (t_96_ = −4.91, *P* ≤ 0.001). As expected, VAT fat mass was significantly different between groups with BMI < 25 and BMI ≥ 25 (Mann–Whitney U = 230, *P* ≤ 0.001 two-tailed). In the group with BMI < 25, after controlling for age, gender and eGFR, VAT fat mass was not significantly associated with UA (t (36) = 1.54, *P* ≥ 0.05, *R* ^2^ = 0.28). However, in the group with BMI ≥ 25, there was a statistically significant positive association between plasma UA and visceral adipose tissue fat mass (t (49) = 2.76, *P* = 0.008, *R* ^2^ = 0.31).

Plasma UA level was significantly different between groups with VAT fat mass ≤ 300 and VAT fat mass > 300 g (t_58.280_ = −5.10, *P* ≤ 0.001). After controlling for age, gender and eGFR, no significant association was observed between plasma UA and VAT fat mass in the group with VAT fat mass ≤ 300 g (t (14) = − 1.54, *P* ≥ 0.05, *R* ^2^ = 0.13). However, VAT fat mass was significantly associated with plasma UA in the group with VAT fat mass > 300 g (t (71) = 3.63, *P* ≤ 0.001, *R* ^2^ = 0.39).

No association was observed between plasma UA and SAT fat mass (*r* (97) = 0.02, *P* ≥ 0.05).

### Associations between plasma UA, plasma TG, TC, LDL-C and HDL-C

A statistically significant positive association was observed between plasma UA and plasma TG (*r*_*s*_ (95) = 0.40, *P* ≤ 0.001). This association remained statistically significant after controlling for age, gender, eGFR and BMI (*t* (88) = 2.30, *P* = 0.024, *R* ^2^ = 0.47), but no longer remained significant after adjustment for VAT fat mass (t (86) = 1.48, *P* ≥ 0.05, *R* ^2^ = 0.44).

There was no association found between plasma UA, TC (*r* (98) = 0.03, *P* ≥ 0.05) and LDL-C (*r* (98) = 0.13, *P* ≥ 0.05).

**Fig. 3:**
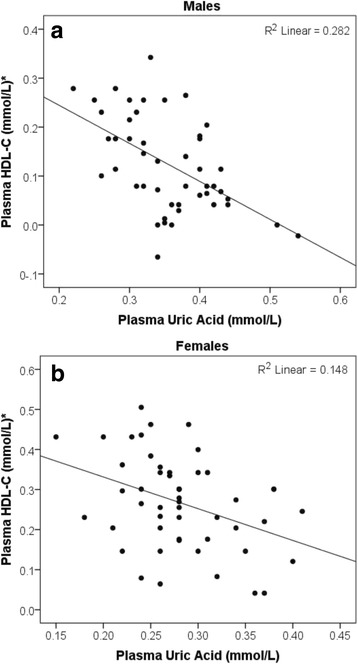
Associations between plasma uric acid and HDL-C in **a** males **b** Females. *Data represented as base 10-log transformed HDL-C

There was a statistically significant negative association between plasma UA and plasma HDL-C (*r* (98) = − 0.61, *P* ≤ 0.001) (Fig. 3). Since the residuals for the linear regression model were not normally distributed when gender was in the equation, the association between plasma UA and HDL-C was analysed separately for each gender. The results showed that after controlling for age, eGFR and BMI, there was a statistically significant negative association between plasma UA and plasma HDL-C in males (t (41) = −2.46, *P* = 0.018, *R* ^2^ = 0.43), but not for females (t (46) = −1.52, *P* ≥ 0.05, *R* ^2^ = 0.25). Also, after controlling for age, eGFR and VAT fat mass, the association remained statistically significant in males (t (39) = −2.16, *P* = 0.037, *R* ^2^ = 0.43), but not for females (t (46) = −1.57, *P* ≥ 0.05, *R* ^2^ = 0.29). After controlling for age, eGFR, BMI and VAT fat mass, no significant association was observed in males (t (38) = −1.99, *P* ≥ 0.05, *R* ^2^ = 0.43) and females (t (45) = −1.43, *P* ≥ 0.05, *R* ^2^ = 0.27).

### Associations between plasma UA and plasma inflammatory markers CRP, TNF- α and IL-6

A statistically significant positive association was observed between plasma UA and plasma CRP (*r*_*s*_ (98) = 0.23, *P* = 0.026). Since the linear regression model did not meet the assumption of normality of residuals, and transformation was not helpful in solving this issue, the association between plasma UA and CRP was analysed separately for each gender. This association remained statistically significant after adjustment for age and eGFR in both males (*t* (41) = 2.55, *P* = 0.015, *R* ^2^ = 0.12) and females (*t* (45) = 3.16, *P* = 0.003, *R* ^2^ = 0.13). It also remained statistically significant after further adjusting for VAT fat mass in females (t (44) = 2.47, *P* ≤ 0.05, *R* ^2^ = 0.37), but not for males (t (38) = 2.13, *P* ≥ 0.05, *R* ^2^ = 0.05). However, the association did not remain significant after adjusting for BMI in the model in either males (*t*(40) = 0.75, *P* ≥ 0.05, *R* ^2^ = 0.20) or females (*t* (44) = 1.471, *P* ≥ 0.05, *R* ^2^ = 0.46). After controlling for age, eGFR, BMI and VAT fat mass, no significant association was observed in males (t (37) = 0.27, *P* ≥ 0.05, R^2^ = 0.11) and females (t (43) = 0.21, *P* ≥ 0.05, R^2^ = 0.48).

No association was observed between plasma UA and other inflammatory markers tested, plasma TNF-α (*r* (97) = 0.12, *P* ≥ 0.05), and plasma Il-6 (*r* (96) = −0.02, *P* ≥ 0.05).

## Discussion

A number of cross-sectional and prospective cohort studies support an association between plasma/serum UA and diabetes risk [2–4]. However, as many diabetes risk factors also closely correlate with UA [24, 25], it is challenging to make a definite conclusion about whether UA is actually an independent risk factor for diabetes. While several studies have shown the association between UA and visceral adiposity [24, 25, 26], no study has yet considered controlling for visceral fat mass. Therefore, in order to improve our understanding of whether UA is an independent risk factor or an otherwise dependent marker for diabetes, we investigated the associations between plasma UA and FPG, HbA1c, lipid profile and inflammatory markers considering BMI and VAT fat mass contribution. To the best of our knowledge, this is the first examination of non-diabetic males and females for the association between UA and diabetes risk factors taking in to account DXA-measured VAT fat mass.

Consistent with previous reports [24] we observed that plasma UA levels were significantly higher in males than females, most likely due to the effect of estrogen on enhancing renal clearance of UA [27]. We also observed a positive association between plasma UA and FPG and HbA1c after adjustment for age, gender and eGFR, consistent with previous observations by others [6, 11, 28, 29]. However, these associations did not remain significant after we adjusted for BMI or VAT fat mass. Similar to our findings, Nan et al. also found the association between UA and glucose did not remain significant after adjustment for BMI [28]. However, in a cross-sectional study in healthy Korean men the reported positive association between serum UA and glucose appeared to be independent of BMI [29]. Though, the potential contribution of VAT fat mass was not assessed in these studies. Another study, reported that even though elevated plasma UA was associated with higher diabetes risk independent of other risk factors such as obesity, the degree of adiposity (BMI and waist circumference) was responsible for a large part of the association between plasma UA and diabetes risk [4]. Similar to our findings, Wei et al., observed a positive association between UA and HbA1c in normoglycemic subjects that did not remain significant after adjustment for a cluster of confounders such as age, sex, and BMI [6].

In the present study, dual-energy X-ray absorptiometry (DXA), was used for the measurement of android to gynoid fat ratio and visceral and subcutaneous adipose tissue. VAT computed by DXA has been validated using CT (computed tomography), the gold standard for visceral fat quantification [30]. It is an inexpensive method with lower radiation dosage. To the best of our knowledge, no study has yet investigated the association between FPG and DXA measured VAT, and android to gynoid fat ratio in non-diabetic middle-aged males and females. Both android fat and gynoid fat measures have been shown to be closely correlated with traditional fat measures like waist circumference and hip circumference, though they are considered more precise indices of the association between fat distribution and metabolic disease [16]. To determine the specific correlates of android fat components, we investigated the associations between VAT (visceral adipose tissue) and SAT (subcutaneouse adipose tissue) fat mass, FPG and plasma UA. Furthermore, for the first time, we used the recently suggested VAT fat mass cut-off value of 300 g as a cut-point for metabolic syndrome risk, in young, healthy and lean women [31], to compare the UA levels in two groups of high and low VAT fat mass values.

Consistent with previous studies [15], we observed significant associations between FPG, plasma TG and BMI. Furthermore, our results showed significant associations between FPG, android to gynoid fat ratio and VAT fat mass, but not SAT fat mass. This observation is supported by previous studies in women [16, 31] and adds further evidence for the value of DXA-measured VAT fat mass and android to gynoid fat ratio to predict the FPG in non-diabetic middle-aged males and females.

Importantly the observed positive association between plasma UA and BMI independent of age, gender and eGFR, did not remain significant after further adjustment for VAT fat mass. However, the positive association between plasma UA and VAT fat mass remained significant after adjustment for age, gender, eGFR and even BMI. The absence of any association between plasma UA and SAT fat mass suggests that visceral fat is a primary driver in the process leading to high plasma UA levels. Our results are consistent with that of others which suggest increased visceral adiposity is a more sensitive predictor of metabolic changes, such as increase in plasma UA levels than the less nuanced physiological measure of BMI [26, 32].

Our data has also shown that android to gynoid fat ratio was significantly associated with UA after adjustment for age, gender, eGFR, BMI, but not VAT fat mass. Similarly, another study reported a positive association between UA and DXA-measured VAT fat area and android to gynoid fat ratio in diabetics. Though, the potential contribution of BMI or VAT fat mass was not assessed in that study [25].

Much of the literature addressing the association between plasma UA and glucose has focused on adjustment for BMI, or waist circumference [12, 13, 33]. However, recent application of DXA has allowed us to investigate this association in more detail for the potential confounding effects of abdominal and visceral fat deposition. Based on findings of our and similar studies [26, 32] it can be suggested that higher visceral fat accumulation, which is also reflected in higher android to gynoid fat ratio, may have a greater influence on the UA metabolism than the less specific BMI measure which includes subcutaneous fat accumulation. While the mechanisms responsible for this association are not completely understood, it has been shown that adipose tissue xanthine oxidoreductase activity can increase UA production in obese mice [34]. Visceral fat has also been shown to be associated more closely with overproduction of UA than subcutaneous fat in obese subjects [32]. VAT may have differential metabolic risks compared to SAT [35]. It is metabolically active and regulates numerous adipocytokines which have been associated with insulin resistance [36]. Insulin resistance or hyperinsulinemia can increase UA reabsorption from the renal tubules, reducing urinary UA excretion and thereby increasing circulating plasma UA levels [37]. Furthermore, increased VAT accumulation causes the over-flow of free fatty acids to the liver and the overproduction of very low-density lipoproteins and TG. The increased need for NADPH during this increase in lipid synthesis accelerates the pentose phosphate pathway which leads to the de novo purine synthesis, thus increasing the production of UA [38, 39]. In our study, the VAT fat mass was significantly and positively associated with the plasma TG levels (data not shown), and the plasma TG levels were positively associated with the plasma UA levels after adjustment for possible confounders such as BMI supporting the relationship between UA production and TG synthesis [24]. Consistent with this hypothesis, another study in normal Japanese men showed that plasma TG was positively associated with plasma UA [26]. More importantly, considering the observed strong associations between VAT fat mass and FPG and also VAT fat mass and UA in our study, it can be suggested that the association between UA and FPG is largely dependent on VAT fat mass which may have direct causal links to both increased plasma UA and increased FPG.

We observed no significant association between plasma UA and TC and LDL-C which was consistent with the findings of others [40]. Our data showed a negative association between UA and HDL-C. However, this association did not remain significant after adjustment for BMI and VAT fat mass. Consistent with our results, a population-based study showed that after adjustment for BMI and waist circumference, the association between UA and HDL-C is lost [41].

A positive association between plasma UA and CRP was observed. However, this association did not remain significant after adjustment for age, eGFR, BMI and VAT fat mass. Similarly, in another study, the association between UA and hsCRP (high-sensitivity CRP) did not remain significant after adjustment for BMI and waist circumference [42]. The individual inflammatory markers of IL-6 and TNF-α, were also not significantly associated with plasma UA as previously reported [43]. Therefore VAT fat mass, rather than UA appears to be a primary driver of inflammation.

While the conclusions in this study are robust, our study had several limitations. First, the cross-sectional nature of the study does not allow us to confirm causality. Also the relatively small number of subjects (*n* = 100) may reduce the sensitivity for identification of relationships with small effect sizes. Finally, it has been previously reported that DXA underestimates abdominal adiposity in individuals with lower abdominal fat, and may overestimate it in individuals with greater abdominal fat [44, 45]. However, the accuracy of VAT measures by DXA has since been verified as accurate by CT in a more recent study [30]. Future studies overcoming these limitations are required to verify the consistency of our observations.

## Conclusion

This study has demonstrated, for the first time, that the associations between plasma UA and diabetes risk factors are largely dependent on VAT fat mass, a specific measure of fat deposition than BMI. Our findings may be clinically relevant in terms of primary preventive strategies for chronic disease especially diabetes which may necessitate the need to monitor high VAT fat mass and the consequent high plasma UA as potential prognostic factors.

## Abbreviations

BMI: Body mass index
CRP: C-reactive protein
DXA: dual-energy X-ray absorptiometry
eGFR: estimated Glomerular filtration rate
ELISA: enzyme-linked immunosorbent assay
FPG: Fasting plasma glucose
HbA1c: glycated hemoglobin A1c
HDL-C: High density lipoprotein cholesterol
IL-6: Interleukin-6
LDL-C: Low density lipoprotein cholesterol
ROI: The region of interest
SAT: Subcutaneous adipose tissue
TC: Total Cholesterol
TG: Triglyceride
TNF-α: Tumor necrosis factor- α
UA: Uric acid
VAT: Visceral adipose tissue
